# Supplementary material for: Fenofibrate Facilitates Post-Active Tuberculosis Infection in Macrophages and is Associated with Higher Mortality in Patients under Long-Term Treatment
Source: J Clin Med. 2020 Jan 25;9(2):337. doi: 10.3390/jcm9020337 (PMC7073736; doi:10.3390/jcm9020337)
Supplement: Supplementary file 1 [file jcm-09-00337-s001.pdf]

Supplementary Table S1. Characteristics of the study cohorts.

Variables	Total (N = 3025)		With Fenofibrate (N = 605)		Without Fenofibrate (N = 2420)		*p* Value *
	No.	%	No.	%	No.	%	
Gender							0.999
Male	2145	70.9	429	70.9	1716	70.9	
Female	880	29.1	176	29.1	704	29.1	
Age (mean ± SD, years)	61.5 ± 16.7		61.3 ± 17.5		61.6 ± 17.7		0.740
Anti-TB medications **	2188	72.3	416	68.8	1772	73.2	0.017
Comorbidities							
Congestive heart failure	139	4.6	24	4.0	115	4.8	0.240
Hypertension	338	11.2	79	13.1	259	10.7	0.080
Diabetes	555	18.4	108	17.9	447	18.5	0.387
COPD	220	7.3	36	6.0	184	7.6	0.093
Emphysema	14	0.5	3	0.5	11	0.5	0.552
Pneumoconiosis	27	0.9	2	0.3	25	1.0	0.071
Chronic kidney disease	124	4.1	23	3.8	101	4.2	0.390
Level of care							0.003
Medical center	1159	38.3	256	42.3	903	37.3	
Regional hospital	1132	37.4	233	38.5	899	37.2	
Local hospital	734	24.3	116	19.2	68	25.5	
Income (NTD)							0.744
<18,000	2.988	98.8	597	98.7	2.391	98.8	
18,000–34,999	29	1.0	7	1.2	22	0.9	
≧35,000	8	0.3	1	0.2	7	0.3	

* Comparisons between the two groups were performed using the Chi-square/Fisher exact test (for categorical variable) and Student *t*-test (for continuous variables). ** Anti-tuberculosis (anti-TB) medications represent any treatment regimen, including treatment with isoniazid and rifampin.

Supplementary Table S2. Cause of death.

Cause	With Fenofibrate (N = 108)		Without Fenofibrate (N = 354)		*p* Value *
	No.	%	No.	%	0.472
Infections, sepsis	41	38.0	131	37.0	
Tuberculosis	8	7.4	19	5.4	
Pneumonia	16	14.8	59	16.7	
Other infections or unknown pathogens	17	15.7	53	15.0	
Respiratory cause	25	23.1	101	28.5	
Cancer	14	13.0	55	15.5	
Circulatory cause	11	10.2	32	9.0	
Gastrointestinal, hepatitis, or biliary cause	10	9.3	21	5.9	
Brain injury	5	4.6	5	1.4	
Renal/urologic cause	2	1.9	5	1.4	
Others	0	0.0	4 **	1.1	

* Comparisons between the two groups were performed using an unpaired *t*-test. ** One burn, one poisoning, one intragenic complication, and one unknown cause.

Supplementary Table S3. Kaplan-Meier curve table for cumulative risk of mortality between the study and comparison cohorts using the log-rank test.

In the Tracking of X Year (s)	With Fenofibrate (N = 605)	Without Fenofibrate (N = 2420)	*p* Value
	Number of mortalities		
1	42	105	0.364
2	51	143	0.254
3	58	178	0.121
4	66	205	0.286
5	71	230	0.304
6	75	254	0.498

7	81	281	0.111
8	94	317	0.078
9	98	329	0.044
10	107	342	0.042
11	108	354	0.035
